# Novel Systemic Inflammatory Indices (SII and SIRI) as Mediators Between BMI and Hearing Loss

**DOI:** 10.1155/mi/2294661

**Published:** 2026-04-30

**Authors:** Hesen Huang, Wenkao Zhou, Yu Du, Yunfei Gao, Yunping Fan

**Affiliations:** ^1^ Department of Otolaryngology, The Seventh Affiliated Hospital, Sun Yat-sen University, Shenzhen, Guangdong, 518107, China, sysu.edu.cn; ^2^ Department of Emergency Medicine, Xiang’an Hospital of Xiamen University, Xiamen, Fujian, 361102, China, xmu.edu.cn; ^3^ Department of Otolaryngology-Head and Neck Surgery, Xiang’an Hospital of Xiamen University, Xiamen, Fujian, 361102, China, xmu.edu.cn

**Keywords:** BMI, HL, inflammatory markers, mediation analyses, NHANES

## Abstract

**Objective:**

This study aimed to examine the associations between body mass index (BMI) and hearing loss (HL), and to explore the mediating role of inflammation using data from the National Health and Nutrition Examination Survey (NHANES).

**Methods:**

A cohort of 5489 participants aged ≥20 years from six NHANES cycles (2005–2012 and 2015–2018) was evaluated. Linear regression analysis was used to assess the relationships between BMI and HL. Three models were developed: (1) the association between BMI and low‐frequency (LF) HL and speech‐frequency (SF) HL; (2) the association between BMI and inflammatory markers (systemic immune‐inflammatory index [SII] and systemic inflammatory response index [SIRI]); and (3) the association between SII/SIRI and LFHL/SFHL. Mediation analyses were conducted to evaluate the role of SII and SIRI in the relationship between BMI and HL.

**Results:**

Among the three groups, participants with higher BMIs exhibited higher values for SII, SIRI, LFHL, and SFHL. Positive correlations were observed between BMI and inflammation, inflammation and HL, and BMI and HL in all models. Mediation analysis revealed that SII mediated 9.87% of the effect of BMI on LFHL and 8.25% on SFHL, while SIRI mediated 17.47% and 16.59% of these effects, respectively. Although modest, these consistent mediating effects suggest inflammation contributes to, but does not wholly account for, the obesity–hearing relationship.

**Conclusion:**

This study indicates that systemic inflammatory markers (SII and SIRI) partially mediate the association between BMI and HL. These findings suggest that inflammation represents a potential mechanistic link in obesity‐related auditory dysfunction. Future longitudinal studies are needed to validate these pathways and inform targeted prevention strategies.

## 1. Introduction

Hearing loss (HL) affects 466 million people globally, projected to reach 900 million by 2050, and ranks as the third leading cause of disability [1, 2]. While aging, noise exposure, and lifestyle factors such as cigarette smoking are established risk factors, metabolic factors——particularly obesity—have emerged as modifiable contributors [3–5]. Identifying and elucidating the risk factors associated with HL, as well as exploring their biomarkers, are crucial for understanding and managing this disease.

Body mass index (BMI) is the most commonly used metric to assess the degree of obesity and is calculated by dividing an individual’s body weight in kilograms by the square of their height in meters. Numerous epidemiologic studies conducted in recent years have demonstrated that elevated BMI, particularly within the obese range and to a lesser extent in the overweight category, is positively associated with HL [6–9]. However, the relationship between overweight status and HL remains inconclusive, as other studies have reported no significant association between these two conditions [10, 11]. New evidence suggests that obesity may affect hearing not only through oxidative stress, hypoxia, and spiral ganglion death, but also through inflammation [9, 12], but the extent and nature of this inflammatory pathway remain incompletely characterized.

Prior investigations of obesity‐associated HL have primarily relied on nonspecific inflammatory markers (e.g., C‐reactive protein), yielding inconsistent results [5, 13–15]. While a recent study has linked SII to hearing function using the National Health and Nutrition Examination Survey (NHANES) data [16], no study to date has examined SIRI, nor has any research comprehensively investigated both SII and SIRI—composite indices integrating platelet, neutrophil, monocyte, and lymphocyte counts—as mediators in the obesity–hearing relationship. It was hypothesized that these novel markers, which capture thromboinflammatory and innate immune pathways, may better elucidate the obesity–hearing relationship than single biomarkers.

Recently, the systemic immune‐inflammation index (SII) and the systemic immune response index (SIRI) have been developed as novel inflammatory markers, incorporating platelets and three leukocyte subtypes [9, 17]. These indices reflect three distinct biological pathways related to thrombosis, inflammatory response, and adaptive immune response, thereby providing a comprehensive assessment of the body’s immune‐inflammatory state. Both SII and SIRI have demonstrated significant predictive value for a wide range of diseases, including cardiovascular disease and cancer [18, 19]. In this study, these two novel inflammatory markers were utilized to investigate their potential roles in the association between BMI and HL.

Therefore, the aims of this study were twofold: (1) to examine the associations between BMI, novel inflammatory markers, and HL and (2) to determine whether these novel inflammatory markers mediate the relationship between BMI and HL. The study hypothesized that (1) obesity promotes a pro‐inflammatory state (elevated SII/SIRI) through adipose tissue‐derived cytokine dysregulation; (2) systemic inflammation may impair cochlear microcirculation and blood–labyrinth barrier (BLB) integrity; and consequently, (3) SII and SIRI partially mediate the association between BMI and frequency‐specific HL. This mediation framework posits inflammation as a modifiable intermediary, supporting targeted anti‐inflammatory interventions for obesity‐related auditory dysfunction.

## 2. Methodologies

### 2.1. Study Population and Study Design

**Figure 1:**
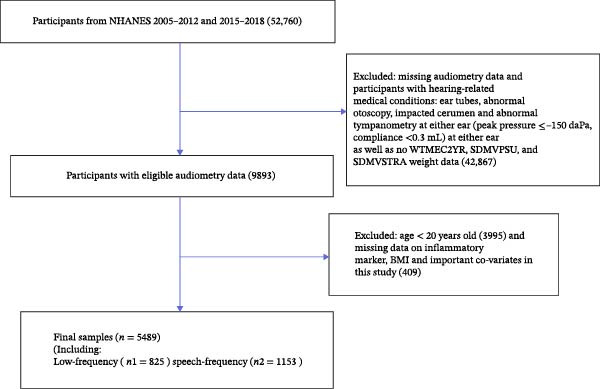
The screening process flowchart for participants.

All participant data for this study were obtained from the NHANES database (2005–2012 and 2015–2018). NHANES is a cross‐sectional, multipurpose research program conducted by the National Center for Health Statistics (NCHS) and the Centers for Disease Control and Prevention (CDC) to assess the health and nutritional status of the civilian, noninstitutionalized population in the United States [20]. Data collection in NHANES is conducted through a combination of questionnaires, physical examinations, and laboratory tests [21]. More information can be found on the NHANES website (https://wwwn.cdc.gov/nchs/nhanes/default.aspx). NHANES is an open dataset approved by the NCHS Ethics Review Board, and all participants provided written informed consent. Given the use of publicly available data, the present study was exempt from additional ethical review by the hospital ethics committee. Inclusion criteria for participants were as follows: (1) age ≥ 20 years and (2) absence of hearing‐related disorders (e.g., ear tubes, abnormalities on otoscopic examination, impacted earwax, and abnormal binaural tympanic chamber pressure measurements [peak pressure ≤ −150 daPa; compliance ≤ 0.3 mL]) [22]. Exclusion criteria included: (1) incomplete hearing or weighting data; (2) missing information on BMI or inflammatory markers; and (3) lack of data on important covariates (e.g., educational level, marital status, smoking status, tinnitus, hypertension, diabetes, dyslipidemia, and moderate physical activity). A detailed flowchart of the participant selection process is shown in Figure 1.

### 2.2. Audiometry

Pure‐tone audiometry (PTA) was used as the outcome variable in this study. Participants underwent standard air‐conducted PTA performed by an experienced and licensed audiologist in a soundproof environment. Binaural hearing thresholds were assessed across a frequency range of 0.5–8 kHz. To ensure the accuracy of participant responses, two 1‐kHz tests were conducted in each ear [22]. The LF hearing PTA was calculated by averaging the hearing thresholds at 0.5, 1, and 2 kHz. The SF hearing PTA was obtained by averaging the thresholds at 0.5, 1, 2, and 4 kHz. The high‐frequency (HF) hearing PTA was computed by averaging the thresholds at 4, 6, and 8 kHz. HL was defined according to the World Health Organization criteria, with a PTA of ≥25 dB HL in the better hearing ear indicating the presence of HL [23]. In this study, HL was expressed as a continuous variable. HFHL was analyzed as a secondary outcome; however, its high prevalence in older adults reduced statistical power for primary mediation analyses.

### 2.3. BMI and Covariates

In this study, BMI was treated as a continuous exposure variable, and participants were stratified into tertiles based on their BMI values for subsequent analyses. Covariates included sex (female and male), age (younger group: 20–39 years; middle‐aged group: 40–59 years; older group: ≥60 years), ethnicity (Mexican American, other Hispanic, non‐Hispanic white, non‐Hispanic black, and other races), education level (<12th grade, highschool graduation, college, or higher), marital status (married/living with a partner, never married/divorced/separated/widowed), poverty‐income ratio (PIR) (<1.3, 1.3–3.5, and >3.5), tinnitus (defined as a positive response to the question, “In the past 12 months, have you been bothered by a ringing, roaring, or buzzing sound in your ears or head that lasted 5 min or more?”), smoking status (defined as having smoked at least 100 cigarettes in one’s lifetime), alcohol consumption (defined as four or more drinks per day), hypertension (defined as a physician diagnosis on two or more visits), diabetes (defined by self‐reported diagnosis and/or current use of insulin or diabetes medications), noise exposure (defined as exposure to occupational, firearm, or recreational noise), dyslipidemia (defined as triglycerides ≥150 mg/dL [1.7 mmol/L], total cholesterol ≥200 mg/dL [5.2 mmol/L], and low‐density lipoprotein cholesterol ≥130 mg/dL [3.4 mmol/L], high‐density lipoprotein cholesterol ≤40 mg/dL [1.0 mmol/L], self‐reported physician diagnosis, or use of cholesterol‐lowering or lipid‐lowering medications), and moderate physical activity (defined as engaging in activities that result in a small increase in respiration or heart rate, such as brisk walking or carrying a light load, for at least 10 consecutive minutes). Specifically, smoking status was included as an essential a priori covariate in our models, given its independent, cross‐sectional association with HL demonstrated in previous NHANES analyses [3].

### 2.4. Measurement of Systemic Inflammatory Indices in Peripheral Blood

Whole blood samples were analyzed at the NHANES Mobile Examination Center. Parameters for whole blood cell counts were derived using the Beckman Coulter counting and sizing method, combined with an automated dilution and mixing device for sample processing and a single‐beam photometer for hemoglobin determinations. Volume, conductivity, and scatter (VCS) technology was used to resolve discrepancies in blood cell counts. Detailed analytical methods can be found on the NHANES website. Four variables were extracted from the NHANES dataset, including lymphocyte count (×1000 cells/µL), monocyte count (×1000 cells/µL), neutrophil count (×1000 cells/µL), and platelet count (×1000 cells/µL). The immunoinflammatory indices were calculated using the following formulas: SII = platelets × neutrophils/lymphocytes, and SIRI = neutrophils × monocytes/lymphocytes [17].

### 2.5. Statistical Analysis

All analyses employed NHANES sample weights (WTMEC2YR, SDMVPSU, and SDMVSTRA) to account for the complex survey design [23]. Continuous variables are reported as weighted means (95% CI) with *p* values from linear regression and categorical variables as weighted percentages (95% CI) with *p* values from *χ* ^2^ tests. Associations were examined in three sequential linear regression models: (1) BMI versus low‐frequency HL (LFHL) and speech‐frequency HL (SFHL); (2) BMI versus SII and SIRI; and (3) SII/SIRI versus LFHL/SFHL. Model 1 was unadjusted; Model 2 adjusted for sex, age, race, marital status, and education; Model 3 further adjusted for PIR, tinnitus, smoking, alcohol, hypertension, diabetes, noise exposure, dyslipidemia, and moderate physical activity. Mediation analysis [24] decomposed the total effect of BMI on LFHL and SFHL into direct and indirect (SII‐ or SIRI‐mediated) effects. Analyses were performed in EmpowerStats 6.0 and SPSS 29.0; two‐sided *p* < 0.05 defined statistical significance.

## 3. Results

### 3.1. Basic Characteristics of the Study Population

Figure 1 outlines participant selection: 5489 adults were screened. A total of 47,271 subjects were excluded, comprising 42,867 individuals with missing weight data, missing hearing data, or hearing‐related conditions, and 4404 subjects lacking BMI, inflammatory marker data, key covariates, or aged under 20 years. Table 1 presents the baseline characteristics of subjects across different BMI tertiles in this study. Supporting Information 1: Table S1 details baseline characteristics across BMI tertiles. The highest tertile showed greater prevalences of alcohol use, tinnitus, hypertension, dyslipidemia, diabetes, and noise exposure, and lower education; similar trends occurred among Mexican Americans, non‐Hispanic Blacks, and middle‐aged adults. SII, SIRI, LFHL, and SFHL were also elevated in tertile 3. Smoking and moderate physical activity did not differ across tertiles (*p* > 0.05).

**Table 1:** Linear regression analysis examines the relationship between BMI and hearing loss among all participants in different models.

Hearing loss	*β* (95% CI), *p*‐t value of PTA level (dB)		
Continuous	Model 1	Model 2	Model 3
LFHL	0.132 (0.088, 0.176) < 0.001	0.069 (0.031, 0.108) < 0.001	0.065 (0.019, 0.111) 0.006
SFHL	0.144 (0.096, 0.193) < 0.001	0.068 (0.028, 0.108) < 0.001	0.053 (0.006, 0.100) 0.027

*Note*: Model 1 is a coarse model with no covariates; the adjusted covariates in model 2 are gender, age, race, education, and marriage; the adjusted covariates in model 3 are gender, age, race, education, marriage, drinking, PIR, tinnitus, noise exposure, BMI, hypertension, dyslipidemia, diabetes, and moderate physical activity. *p*‐t, *p* for trend. Weighted by WTMEC2YR.

Abbreviations: CI, confidence interval; OR, odds ratio.

### 3.2. The Relationship Between BMI and HL

Table 1 presents the associations between BMI and HL among all participants, as assessed using linear regression models. In Model 1, BMI was significantly and positively associated with both LFHL (*β* = 0.132, 95% CI = 0.088–0.176, *p* < 0.001) and SFHL (*β* = 0.144, 95% CI = 0.096–0.193, *p* < 0.001). In Model 2, after adjusting for a subset of covariates, BMI remained significantly and positively correlated with LFHL (*β* = 0.069, 95% CI = 0.031–0.108, *p* < 0.001) and SFHL (*β* = 0.068, 95% CI = 0.028–0.108, *p* < 0.001). In Model 3, after adjusting for all covariates, BMI continued to exhibit significant positive associations with both LFHL (*β* = 0.065, 95% CI = 0.019–0.111, *p* = 0.006) and SFHL (*β* = 0.053, 95% CI = 0.006–0.100, *p* = 0.027).

### 3.3. Inflammatory Markers Associated With BMI and HL

Table 2 presents the associations between BMI and the inflammatory markers SII and SIRI, analyzed using linear regression models for all participants. In Model 1, BMI was positively correlated with both SII (*β* = 4.790, 95% CI = 3.681–5.899, *p* < 0.001) and SIRI (*β* = 0.011, 95% CI = 0.008–0.014, *p* < 0.001). In Model 2, BMI remained significantly and positively associated with SII (*β* = 4.389, 95% CI = 3.281–5.498, *p* < 0.001) and SIRI (*β* = 0.010, 95% CI = 0.007–0.012, *p* < 0.001). In Model 3, after adjusting for all covariates, BMI continued to exhibit significant positive associations with both SII (*β* = 4.285, 95% CI = 2.928–5.641, *p* < 0.001) and SIRI (*β* = 0.007, 95% CI = 0.004–0.011, *p* < 0.001). Table 3 examines the relationships between the inflammatory markers (SII and SIRI) and HL (LFHL and SFHL) using the same methodology. In Models 1, 2, and 3, SII showed significant positive correlations with both LFHL and SFHL (*p* < 0.001). Similarly, SIRI was significantly and positively associated with LFHL and SFHL across all models (*p* < 0.001).

**Table 2:** Linear regression analysis examines the relationship between BMI and SII/SIRI among all participants in different models.

Inflammatory markers	*β* (95% CI), *p*‐t value		
Continuous	Model 1	Model 2	Model 3
SII	4.790 (3.681, 5.899) < 0.001	4.389 (3.281, 5.498) < 0.001	4.285 (2.928, 5.641) < 0.001
SIRI	0.011 (0.008, 0.014) < 0.001	0.010 (0.007, 0.012) < 0.001	0.007 (0.004, 0.011) < 0.001

*Note*: Model 1 is a coarse model with no covariates; the adjusted covariates in model 2 are gender, age, race, education, and marriage; the adjusted covariates in model 3 are gender, age, race, education, marriage, drinking, PIR, tinnitus, noise exposure, BMI, hypertension, dyslipidemia, diabetes, and moderate physical activity. *p*‐t, *p* for trend. Weighted by WTMEC2YR.

Abbreviations: CI, confidence interval; OR, odds ratio.

**Table 3:** Linear regression analysis examines the relationship between SII, SIRI, and hearing loss among all participants in different models.

SII	*β* (95% CI), *p*‐t value of PTA level (dB)		
Continuous	Model 1	Model 2	Model 3
LFHL	0.004 (0.003, 0.005) < 0.001	0.002 (0.002, 0.003) < 0.001	0.002 (0.001, 0.003) < 0.001
SFHL	0.004 (0.003, 0.005) < 0.001	0.003 (0.002, 0.004) < 0.001	0.002 (0.001, 0.003) < 0.001

**SIRI**	***β* (95% CI), P-t value of PTA level, dB**		

Continuous	Model 1	Model 2	Model 3
LFHL	1.848 (1.459, 2.238) < 0.001	1.082 (0.738, 1.426) < 0.001	0.914 (0.527, 1.301) < 0.001
SFHL	2.304 (1.872, 2.735) < 0.001	1.190 (0.835, 1.545) < 0.001	0.975 (0.578, 1.371) < 0.001

*Note*: Model 1 is a coarse model with no covariates; the adjusted covariates in model 2 are gender, age, race, education, and marriage; the adjusted covariates in model 3 are gender, age, race, education, marriage, drinking, PIR, tinnitus, noise exposure, BMI, hypertension, dyslipidemia, diabetes, and moderate physical activity. *p*‐t, *p* for trend. Weighted by WTMEC2YR.

Abbreviation: CL, confidence interval.

### 3.4. Mediating Role of Inflammatory Markers in the Association Between BMI and LFHL, SFHL

**Figure 2:**
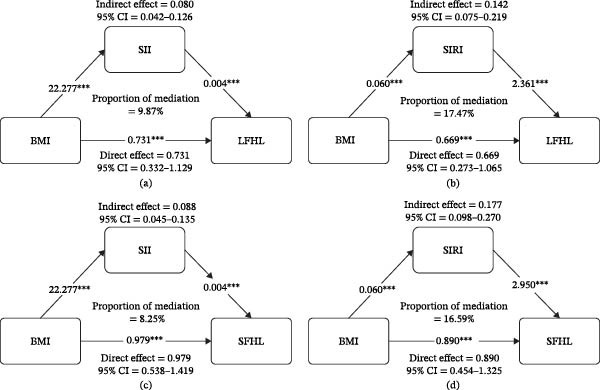
Correlation analysis of BMI mediated by novel systemic inflammatory markers SII and SIRI with LFHL and SFHL.

Figure 2 and Supporting Information 2: Table S2 show that the 95% bootstrap CIs for the indirect effects of BMI on LFHL and SFHL via SII and SIRI excluded zero, confirming significant mediation. BMI exhibited both direct and indirect effects, satisfying criteria for partial mediation (significant total and indirect effects with positively proportional mediation) [25]. LFHL: total effect = 0.812 (95% CI 0.411–1.210, *p* < 0.001); indirect effects via SII = 0.080 (95%CI 0.042–0.126) and via SIRI = 0.142 (95% CI 0.076–0.220), accounting for 9.87% and 17.47% of the total effect, respectively; direct effect = 0.731 (95% CI 0.332–1.129, *p* < 0.001). SFHL: SII mediated 8.25% and SIRI mediated 16.59% of the BMI–SFHL relationship. The results indicate that systemic inflammation, reflected by SII and SIRI, substantially mediates the association between BMI and HL.

## 4. Discussion

This study confirmed that both BMI and systemic inflammation independently predict HL. Participants in the highest BMI tertile exhibited significantly elevated SII and SIRI levels, consistent with prior reports linking adiposity to chronic inflammation [26]. After full adjustment (Model 3), BMI remained positively associated with both inflammatory markers, reinforcing the robustness of this relationship [26, 27]. These data indicate that systemic inflammation partially mediates the BMI–HL association, underscoring the need to address obesity and inflammatory pathways in HL prevention strategies.

HL can be categorized by frequency into LFHL, SFHL, and HFHL [28]. HF audiometry is primarily used for the early detection of HL. Age‐related HL in older adults typically begins with HFHL and progressively affects the lower and middle frequencies. Audiometry in the 2–4 kHz range is particularly important for assessing speech comprehension [29]. The prevalence of HFHL among participants aged ≥60 years was 89.98%, consistent with prior literature reporting 95.07% in adults aged ≥70 years [30]. Due to this high prevalence in older adults, HFHL was analyzed as a secondary outcome only. Age is a well‐recognized risk factor for HL because hearing tends to deteriorate progressively with age. Therefore, LFHL and SFHL were selected as the primary outcomes of the present study. Cross‐sectional studies have reported that elevated BMI is associated with poorer hearing thresholds [1, 31, 32]. A meta‐analysis previously noted a linear relationship between BMI and HL, supporting a positive association between elevated BMI and HL [1]. Similarly, a study in an adolescent population found a positive association between obesity and prevalent HL [33, 34]. These findings are consistent with the present results, which demonstrated that adults in the higher tertiles of BMI exhibited elevated values of LFHL and SFHL.

Acute‐phase biomarkers, including hsCRP and white blood cell count, have been linked to HL [13, 14]. In a U.S. cohort, adults with sustained hsCRP >3 mg/L or rising levels over 10 years exhibited approximately double the risk of HL compared with those with hsCRP <1 mg/L [13]. Wang et al. [15] showed an association between glycoprotein acetylation, an emerging biomarker of chronic and cumulative inflammation, and poor hearing during mid‐childhood and middle age. However, a study by Shruti Gupta et al. [5] found no statistically significant associations between HL and the inflammatory plasma markers CRP, IL‐6, and TNFR‐2. Nonetheless, the lack of a clear association in these studies does not necessarily imply the absence of a link between inflammatory markers and HL; rather, these markers may not be the most suitable indicators to validate this relationship. Beyond these traditional markers, recent literature has increasingly highlighted the prognostic utility of various hematological parameters in auditory health. For instance, Zhang et al. [35] demonstrated significant correlations between routine blood parameters and the recovery outcomes of sudden sensorineural HL. Similarly, other composite inflammatory indices—such as the neutrophil‐to‐lymphocyte ratio (NLR), the SII, and the pan‐immune‐inflammation value (PIV)—have been suggested as valuable prognostic tools for evaluating the inflammatory burden in clinical auditory disorders [36]. In this study, monocyte, platelet, neutrophil, and lymphocyte counts were utilized to construct two novel inflammatory indices. SII (platelet × neutrophil/lymphocyte) reflects thromboinflammatory status, combining platelet‐mediated microvascular dysfunction with neutrophil‐predominant innate immunity. SIRI (neutrophil × monocyte/lymphocyte) captures monocyte–macrophage axis activation, a key pathway in chronic metabolic inflammation. These composite indices offer mechanistic specificity beyond single biomarkers [17]. In Models 1, 2, and 3, it was consistently observed that SII exhibited a significant positive correlation with LFHL and SFHL. Similarly, SIRI showed a statistically significant positive correlation with LFHL and SFHL. This aligns with recent literature demonstrating a positive association between elevated SII levels and HL in the general adult population [16].

The observed associations between SII/SIRI and HL may reflect specific pathophysiological processes that connect systemic inflammation to cochlear vulnerability. SII integrates platelet count, which regulates thrombosis and microcirculatory perfusion, with NLR, a marker of innate‐to‐adaptive immune balance. Elevated SII suggests a pro‐thrombotic, pro‐inflammatory state that may compromise the cochlea’s terminal vascular supply. The cochlea is particularly susceptible to ischemic injury due to its end‐arterial blood supply from the labyrinthine artery without collateral circulation [37]. Platelet activation in obesity‐related inflammation releases thromboxane A2 and platelet‐derived growth factor, promoting microvascular stasis and hypoxia in the stria vascularis [38]. SIRI, incorporating monocyte count, captures monocyte–macrophage axis activation—a central pathway in obesity‐induced adipose tissue inflammation. Circulating monocytes infiltrate adipose tissue and differentiate into pro‐inflammatory M1 macrophages, sustaining systemic cytokine release (IL‐1β, IL‐6, and TNF‐α) [39]. These cytokines can: (1) increase blood viscosity and reduce cochlear blood flow velocity; (2) upregulate adhesion molecules (ICAM‐1 and VCAM‐1) on cochlear capillary endothelium, facilitating leukocyte margination and capillary occlusion; and (3) directly damage outer hair cells through oxidative stress‐mediated mitochondrial dysfunction [40–42]. Crucially, the BLB, formed by tight junctions between capillary endothelial cells and pericytes in the stria vascularis, restricts large‐molecule entry into the endolymph. Systemic inflammation disrupts BLB integrity through multiple converging pathways: (i) TNF‐α and IL‐1β induce endothelial cytoskeletal reorganization and tight junction protein (occludin and claudin‐5) downregulation; (ii) neutrophil extracellular traps (NETs) released during heightened neutrophil activity (reflected in elevated SII) promote endothelial cytotoxicity; and (iii) monocyte‐derived matrix metalloproteinases (MMP‐2 and MMP‐9) degrade basement membrane components [43, 44]. BLB compromise leads to endolymphatic ionic dysregulation (elevated K+ and reduced Na+), disrupting the endocochlear potential required for hair cell mechanoelectrical transduction [45]. This mechanistic framework positions SII and SIRI not merely as surrogate markers of general inflammation but as indicators of specific pathophysiological processes—thromboinflammation and innate immune activation—that have direct relevance to cochlear microcirculatory and barrier function. Increased inflammation may directly disrupt the integrity of vascular endothelial cells in the stria vascularis, a highly vascularized region of the cochlea. This disruption can lead to the rupture of the BLB, thereby affecting the electrical activity of hair cells and the function of the auditory nerve [46].

According to the literature, inflammation mediates a substantial proportion of the associations between obesity and HL. Glycoprotein acetylation has been reported to account for 11%–53% of the total effect of obesity on HL in adults and 53%–67% in children, while hsCRP has shown a less pronounced but similar mediating role [47]. In contrast, the present study not only categorized HL in detail but also, for the first time, constructed two novel inflammatory markers (SII and SIRI) based on a comprehensive panel of blood cells to investigate their mediating roles in the relationship between BMI and different types of HL. It was found that BMI‐mediated effects on LFHL were partially mediated by SII (9.87%) and SIRI (17.47%). Similarly, the association between BMI and SFHL was mediated by SII (8.25%) and SIRI (16.59%). These findings highlight the potential role of systemic inflammation in the relationship between BMI and HL. The biological mechanisms underlying the contribution of excess weight to HL remain unclear. Adipose tissue functions as an endocrine organ, secreting a wide range of pro‐ and anti‐inflammatory adipokines [48]. Excessive obesity promotes a pro‐inflammatory state, characterized by the downregulation of anti‐inflammatory adipokines and the upregulation of pro‐inflammatory adipokines. This imbalance can lead to vascular dysfunction and end‐organ damage, including cochlear damage, in the context of HL [49–51]. Lipocalin, an anti‐inflammatory adipokine, plays a crucial role in regulating metabolism, insulin sensitivity, inflammation, and atherosclerosis [52]. Obesity is associated with low plasma lipocalin levels [53]. Elevated plasma lipocalin levels have been shown to be negatively correlated with HL in humans, suggesting that low plasma lipocalin levels may contribute to the development of HL [1, 54]. Cox et al. [55] proposed that alterations in the gut microbiota and intestinal permeability may trigger inflammation in obesity, potentially informing the mechanisms of HL in the general population.

The principal strength of the present study rests on the use of NHANES, a large, nationally representative dataset that enhances the external validity of our conclusions. This study also controlled for an extensive set of sociodemographic and lifestyle covariates, increasing confidence in the observed associations. Moreover, by introducing SII and SIRI—novel, blood cell‐based inflammatory indices—as potential mediators, the first systematic evidence is provided linking BMI‐related systemic inflammation to LFHL and SFHL. Nevertheless, several limitations should be acknowledged. First, the high exclusion rate has raised concerns about selection bias. Participants were mainly excluded due to missing hearing tests, abnormal otoscopy, or incomplete blood marker data. Although direct comparison between included and excluded participants is not feasible, excluded individuals may have greater comorbidities or mobility impairments, indicating that our research findings may underestimate the true association and best generalize to relatively healthy adults. Second, the cross‐sectional design fundamentally limits causal inference for the mediation analysis. Specifically, temporal ordering among BMI, inflammatory markers, and HL cannot be established; unmeasured confounding (e.g., genetic susceptibility, cumulative noise exposure, and dietary quality) may bias effect estimates; and reverse causation (e.g., early HL reducing physical activity and increasing BMI) cannot be excluded. Consequently, the reported mediation proportions (8%–17%) should be interpreted as exploratory rather than confirmatory evidence of inflammatory pathways. Third, despite comprehensive adjustment, residual or unmeasured confounding (e.g., genetic factors and detailed dietary data) cannot be excluded. Fourth, certain variables (e.g., physical activity and noise exposure) were self‐reported and may be subject to misclassification. Finally, although NHANES is broadly representative, extrapolation to non‐U.S. populations or to individuals with multiple comorbidities should be approached cautiously and warrants further investigation. Future longitudinal studies should: (1) establish the temporal sequence of obesity, inflammation, and hearing decline; (2) incorporate repeated measures of SII/SIRI to capture inflammatory trajectories; (3) evaluate whether weight loss or anti‐inflammatory interventions can modify hearing trajectories; and (4) explore ear‐specific biomarkers (e.g., perilymph cytokines) to confirm local inflammatory mechanisms. Mendelian randomization approaches may help disentangle causal relationships from confounding.

## 5. Conclusion

This study demonstrate that BMI, systemic inflammation, and HL are intercorrelated, with SII and SIRI partially mediating the BMI–LFHL/SFHL associations. These findings suggest inflammation as a potential mechanistic link in obesity‐related HL and underscore the need for longitudinal studies to confirm susceptibility and to inform targeted preventive strategies.

## Author Contributions

**Hesen Huang:** writing – original draft, investigation, conceptualization, visualization, formal analysis. **Wenkao Zhou, Yu Du, and Yunfei Gao:** writing – original draft, investigation, software. **Yunping Fan:** resources, writing – review and editing, project administration, supervision.

## Funding

This work was sponsored by the National Key R&D Program of China (Grant 2023YFC2410200) and The Shenzhen Basic Research Program (General Program) (Grant JCYJ20250604143259004).

## Ethics Statement

The study was approved by the National Center for Health Statistics (NCHS) Ethics Review Board and written consent was obtained from the subjects.

## Consent

All authors have agreed to the publication.

## Conflicts of Interest

The authors declare no conflicts of interest.

## Supporting Information

Additional supporting information can be found online in the Supporting Information section.

## Supporting information


**Supporting Information 1** Table S1: The demographic and clinical characteristics of the patients by tertiles of baseline BMI.

**Supporting Information 2** Table S2: Decomposition table of total effect, direct effect, and mediating effect.

## Data Availability

The data used in this study are available on the National Health and Nutrition Examination Survey website: https://www.cdc.gov/nchs/nhanes/index.htm.
