# Supplementary material for: Novel Systemic Inflammatory Indices (SII and SIRI) as Mediators Between BMI and Hearing Loss
Source: Mediators Inflamm. 2026 Apr 30;2026:2294661. doi: 10.1155/mi/2294661 (PMC13129582; doi:10.1155/mi/2294661)
Supplement: Supplementary file 1 — Supporting Information 1 Table S1: The demographic and clinical characteristics of the patients by tertiles of baseline BMI. [file MI-2026-2294661-s002.docx]

Supplemental Table 1. The demographic and clinical characteristics of the patients by tertiles of baseline BMI

| Variables | BMI Tertiles | | | p |
| --- | --- | --- | --- | --- |
|  | Q1 (13.180-25.420) | Q2 (25.500-30.860) | Q3 (30.870-82.100) |  |
| Gender |  |  |  | <0.0001 |
| Female | (967) 57.29 (54.79, 59.75) | (851) 47.11 (43.69, 50.57) | (1092) 56.21 (53.51, 58.87) |  |
| Male | (849) 42.71 (40.25, 45.21) | (992) 52.89 (49.43, 56.31) | (738) 43.79 (41.13, 46.49) |  |
| Age (years) |  |  |  | <0.0001 |
| Young (20-39) | (845) 48.93 (43.96, 53.91) | (637) 35.88 (32.86, 39.03) | (618) 34.74 (31.20, 38.46) |  |
| Middle-aged (40-59) | (514) 32.67 (28.36, 37.29) | (633) 39.73 (36.37, 43.19) | (705) 42.64 (39.03, 46.32) |  |
| Older (≥60) | (457) 18.41 (15.13, 22.21) | (573) 24.38 (21.69, 27.29) | (507) 22.62 (19.73, 25.81) |  |
| Race |  |  |  | <0.0001 |
| Mexican American | (162) 5.89 (4.29, 8.03) | (299) 9.44 (6.85, 12.86) | (304) 10.35 (7.14, 14.78) |  |
| Other Hispanic | (156) 4.85 (3.20, 7.27) | (222) 7.09 (5.27, 9.49) | (215) 6.74 (4.87, 9.26) |  |
| Non-Hispanic White | (716) 69.78 (64.56, 74.53) | (755) 68.99 (63.59, 73.92) | (657) 64.10 (57.75, 70.00) |  |
| Non-Hispanic Black | (293) 7.36 (5.48, 9.82) | (346) 8.66 (6.60, 11.30) | (544) 14.34 (10.64, 19.05) |  |
| Other Races | (489) 12.12 (9.68, 15.09) | (221) 5.81 (4.47, 7.54) | (110) 4.47 (3.27, 6.08) |  |
| Educational level |  |  |  | 0.0004 |
| Less than high school | (311) 11.38 (8.67, 14.79) | (425) 14.13 (11.58, 17.13) | (404) 14.96 (12.30, 18.09) |  |
| High school or GED | (314) 15.78 (13.24, 18.69) | (360) 18.55 (15.62, 21.89) | (433) 22.46 (20.03, 25.10) |  |
| Above high school | (1191) 72.84 (67.62, 77.50) | (1058) 67.32 (62.89, 71.46) | (993) 62.58 (58.36, 66.61) |  |
| Marital status |  |  |  | 0.0078 |
| Married/living with partner | (958) 61.67 (57.80, 65.40) | (1147) 67.15 (64.30, 69.88) | (1234) 64.86 (60.85, 68.68) |  |
| Never married/divorced/ separated/widowed | (671) 38.33 (34.60, 42.20) | (642) 32.85 (30.12, 35.70) | (837) 35.14 (31.32, 39.15) |  |
| Alcohol |  |  |  | 0.0131 |
| No | (1248) 73.81 (69.66, 77.57) | (1271) 73.16 (70.18, 75.94) | (1224) 70.09 (67.15, 72.87) |  |
| Yes | (186) 10.25 (8.13, 12.85) | (250) 12.84 (10.54, 15.55) | (244) 15.28 (13.18, 17.65) |  |
| NA | (382) 15.94 (13.23, 19.09) | (322) 14.00 (11.97, 16.31) | (362) 14.63 (12.64, 16.87) |  |
| Smoking |  |  |  | 0.1919 |
| No | (1128) 60.14 (55.75, 64.37) | (1100) 60.78 (57.86, 63.62) | (1088) 56.29 (52.62, 59.90) |  |
| Yes | (688) 39.86 (35.63, 44.25) | (743) 39.22 (36.38, 42.14) | (742) 43.71 (40.10, 47.38) |  |
| PIR |  |  |  | 0.0330 |
| >3.5 | (602) 42.60 (36.96, 48.44) | (580) 44.63 (40.15, 49.19) | (451) 37.23 (31.97, 42.81) |  |
| 1.3-3.5 | (628) 32.66 (29.24, 36.27) | (609) 31.54 (27.63, 35.73) | (676) 35.54 (31.70, 39.57) |  |
| <1.3 | (445) 18.44 (15.57, 21.71) | (513) 18.57 (16.05, 21.39) | (571) 22.04 (18.75, 25.72) |  |
| NA | (141) 6.30 (4.76, 8.29) | (141) 5.26 (4.12, 6.68) | (132) 5.19 (3.94, 6.81) |  |
| Tinnitus |  |  |  | 0.0009 |
| No | (1607) 86.48 (83.82, 88.77) | (1560) 83.76 (81.29, 85.96) | (1477) 80.21 (77.04, 83.04) |  |
| Yes | (209) 13.52 (11.23, 16.18) | (283) 16.24 (14.04, 18.71) | (353) 19.79 (16.96, 22.96) |  |
| Noise exposure |  |  |  | 0.0016 |
| No | (953) 44.98 (41.24, 48.78) | (814) 37.85 (34.03, 41.82) | (818) 37.05 (33.01, 41.28) |  |
| Yes | (863) 55.02 (51.22, 58.76) | (1029) 62.15 (58.18, 65.97) | (1012) 62.95 (58.72, 66.99) |  |
| Hypertension |  |  |  | <0.0001 |
| No | (1339) 75.54 (71.99, 78.77) | (1165) 65.82 (62.53, 68.97) | (1032) 60.52 (58.07, 62.93) |  |
| Yes | (477) 24.46 (21.23, 28.01) | (678) 34.18 (31.03, 37.47) | (798) 39.48 (37.07, 41.93) |  |
| Abnormal blood lipids |  |  |  | <0.0001 |
| No | (973) 54.12 (50.70, 57.49) | (695) 36.63 (33.24, 40.14) | (635) 30.94 (28.31, 33.70) |  |
| Yes | (843) 45.88 (42.51, 49.30) | (1148) 63.37 (59.86, 66.76) | (1195) 69.06 (66.30, 71.69) |  |
| Diabetes |  |  |  | <0.0001 |
| No | (1715) 96.71 (95.38, 97.67) | (1653) 93.57 (92.26, 94.67) | (1492) 84.69 (82.27, 86.82) |  |
| Yes | (101) 3.29 (2.33, 4.62) | (190) 6.43 (5.33, 7.74) | (338) 15.31 (13.18, 17.73) |  |
| Moderate activity |  |  |  | 0.4598 |
| No | (1194) 59.52 (55.23, 63.68) | (1149) 56.86 (53.73, 59.94) | (1116) 57.45 (53.65, 61.15) |  |
| Yes | (622) 40.48 (36.32, 44.77) | (694) 43.14 (40.06, 46.27) | (714) 42.55 (38.85, 46.35) |  |
| SII | (1816) 494.14 (472.77, 515.51) | (1843) 509.81 (493.28, 526.33) | (1830) 547.34 (526.94, 567.75) | <0.0001 |
| SIRI | (1816) 1.11 (1.05, 1.17) | (1843) 1.20 (1.14, 1.25) | (1830) 1.24 (1.19, 1.30) | 0.0001 |
| LFHL (continuous) | (1816) 11.31 (10.43, 12.19) | (1843) 13.19 (12.41, 13.98) | (1830) 14.04 (13.32, 14.76) | <0.0001 |
| SFHL (continuous) | (1816) 13.30 (12.28, 14.32) | (1843) 15.95 (15.07, 16.82) | (1830) 16.53 (15.69, 17.36) | <0.0001 |

P values from χ2 test or Wilcoxon rank-sum test (categorical variables) and Student's t-tests (continuous variables) . Data in the table: For continuous variables: (N-observe) survey-weighted mean (95% CI) , P-value was by survey-weighted linear regression (svyglm) ; For categorical variables: (N-observe) survey-weighted percentage (95% CI) , P-value was by survey-weighted Chi-square test (svytable) .
