# Supplementary material for: Novel Systemic Inflammatory Indices (SII and SIRI) as Mediators Between BMI and Hearing Loss
Source: Mediators Inflamm. 2026 Apr 30;2026:2294661. doi: 10.1155/mi/2294661 (PMC13129582; doi:10.1155/mi/2294661)
Supplement: Supplementary file 2 — Supporting Information 2 Table S2: Decomposition table of total effect, direct effect, and mediating effect. [file MI-2026-2294661-s001.docx]

Supplemental Table 2.Decomposition table of total effect, direct effect, and mediating effect

1. Intermediary factors: SII. Outcome variable:LFHL.

|  | effect value | se | LLCI | ULCI | effect size |
| --- | --- | --- | --- | --- | --- |
| total effect | 0.812 | 0.204 | 0.411 | 1.210 |  |
| direct effect | 0.731 | 0.203 | 0.332 | 1.129 | 90.13% |
| mediation effect | 0.080 | 0.022 | 0.042 | 0.126 | 9.87% |

1. Intermediary factors: SIRI. Outcome variable:LFHL.

|  | effect value | se | LLCI | ULCI | effect size |
| --- | --- | --- | --- | --- | --- |
| total effect | 0.811 | 0.204 | 0.411 | 1.210 |  |
| direct effect | 0.669 | 0.202 | 0.273 | 1.065 | 82.53% |
| mediation effect | 0.142 | 0.036 | 0.075 | 0.219 | 17.47% |

1. Intermediary factors: SII. Outcome variable:SFHL.

|  | effect value | se | LLCI | ULCI | effect size |
| --- | --- | --- | --- | --- | --- |
| total effect | 1.067 | 0.225 | 0.625 | 1.508 |  |
| direct effect | 0.979 | 0.225 | 0.538 | 1.419 | 91.75% |
| mediation effect | 0.088 | 0.023 | 0.045 | 0.135 | 8.25% |

1. Intermediary factors: SIRI. Outcome variable:SFHL.

|  | effect value | se | LLCI | ULCI | effect size |
| --- | --- | --- | --- | --- | --- |
| total effect | 1.067 | 0.225 | 0.625 | 1.508 |  |
| direct effect | 0.890 | 0.222 | 0.454 | 1.325 | 83.41% |
| mediation effect | 0.177 | 0.044 | 0.098 | 0.270 | 16.59% |
